# Synergistic effect of novel CS/N-TiO₂/NT coating and low temperature in prolonging the storage life of *Stropharia rugosoannulata*

**DOI:** 10.1016/j.fochx.2026.104290

**Published:** 2026-08-06

**Authors:** Xingjun Lu, Kun Qiao, Xinyan Liu, Xiaozhen Peng, Bangzhu Peng

**Affiliations:** a College of Laboratory Medicine, Hunan University of Medicine, Huaihua 418000, Hunan, China; b Hunan Provincial Research and Innovation Platform for Universities: Decoding of Novel Biomolecules in Liver Cancer and Intelligent Diagnosis, Huaihua 418000, Hunan, China; c College of Food Science and Technology, Huazhong Agricultural University, No. 1 Shizishan Street, Hongshan District, Wuhan 430070, Hubei, China

**Keywords:** *Stropharia rugosoannulata*, Postharvest treatments, Nano-coatings, Storage temperature

## Abstract

Postharvest *Stropharia rugosoannulata* deteriorates from fungal infection and active metabolism. A novel chitosan/nitrogen doped titanium dioxide/natamycin (CS/N TiO₂/NT) composite film was developedinhibiting Fusarium pseudoanthophilum and Talaromyces purpureogenus by 71.30% and 75.04%, with 132.20% higher tensile strength and 69.29% lower oxygen permeability. Preservation efficacy was markedly temperature dependent. At 25 °C, only marginal improvements were observed. At 10 °C, the coating significantly reduced total colony counts and modulated enzymatic activities, notably activating peroxidase, retarding the decline of superoxide dismutase (SOD), and inhibiting polyphenol oxidase. Optimal preservation was achieved at 4 °C, where respiratory intensity and microbial proliferation were suppressed while SOD activity was effectively sustained. Cox proportional hazards analysis further confirmed that coated samples stored at 4 °C exhibited a 33.33%–38.46% extension in storage life relative to uncoated controls. These findings establish a temperature responsive preservation strategy and provide a mechanistic framework for extending the shelf life of perishable mushrooms.

## Highlights

• A novel CS/N-TiO₂/NT composite film was developed for mushroom preservation.
• The film showed enhanced mechanical strength and reduced oxygen permeability.
• Triple-mechanism antifungal activity achieved broad-spectrum spoilage control.
• Preservation mechanism was temperature-dependent, with optimal effect at 4 °C.
• Storage life was extended by33.33%–38.46% at 4 °C based on Cox model analysis.

## Graphical abstract

**Figure:**
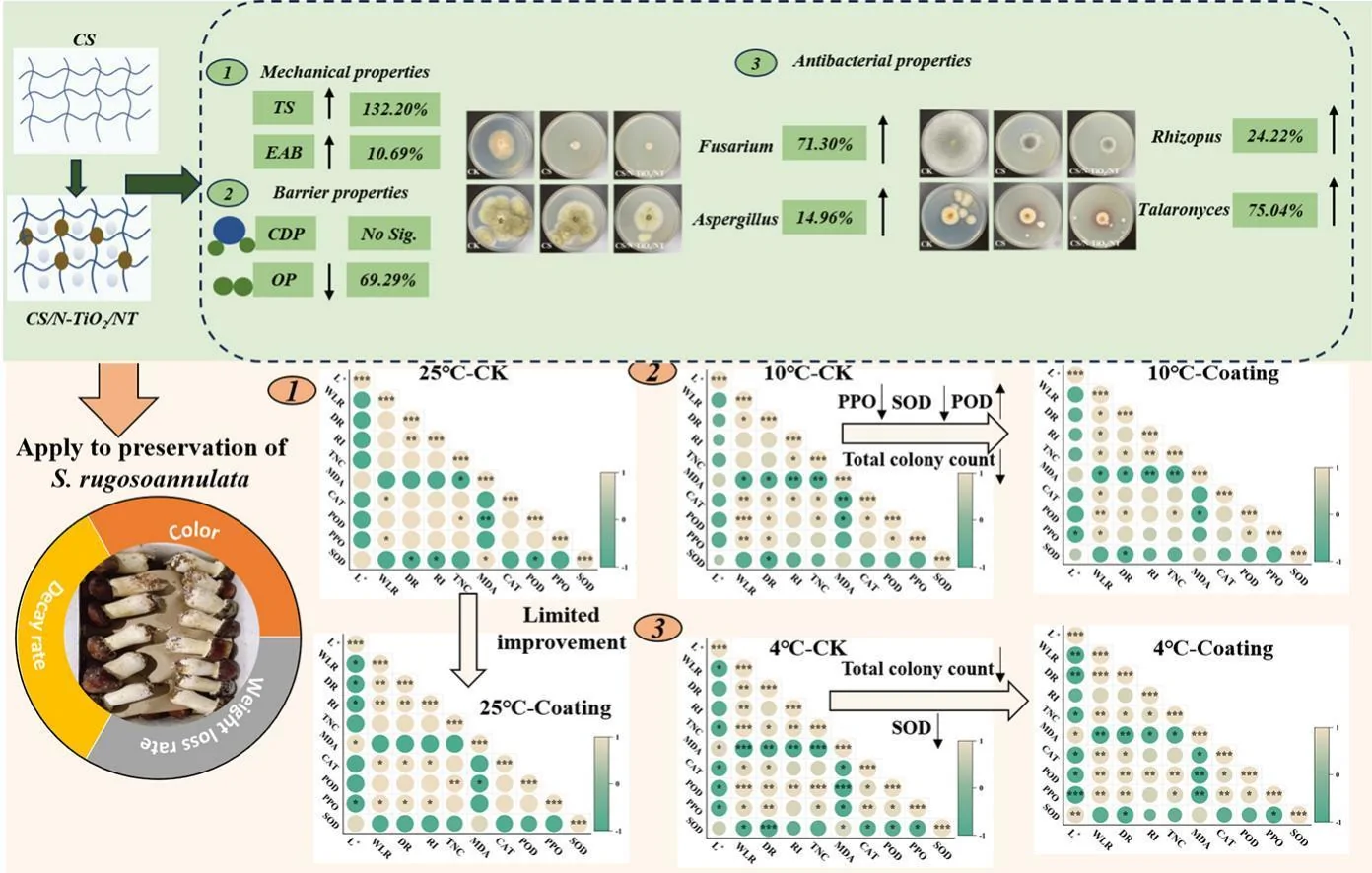

## 1 Introduction

*Stropharia rugosoannulata* (*S. rugosoannulata*) ranks among the top ten internationally traded edible mushrooms (Xue et al., 2024), with its high valuation attributable to a combination of delicate flavor, an exceptional nutritional profile, and notable medicinal properties (Liu et al., 2024). Its recognition by the Food and Agriculture Organization (FAO) as a suitable crop for cultivation in developing countries (Askar et al., 2025) has contributed to a steady expansion of its cultivation area in recent years (Yu et al., 2026). Nevertheless, the commercialization of fresh *S. rugosoannulata* is severely constrained by its inherent perishability. Postharvest deterioration is characterized by pileus discoloration, rupture, stipe shrinkage, and the emergence of white flocculent mold (Zhang et al., 2025). The situation is further complicated by resilient spoilage fungi, including *Fusarium pseudoanthophilum*, *Aspergillus niger*, *Rhizopus azygosporus*, and *Talaromyces purpureogenus* (Liu et al., 2024). Although a range of preservation strategies, such as short-term anaerobic treatment (Liu et al., 2024) and appropriate sub-freezing temperatures (Xue et al., 2024), has been explored, effective preservation technologies specifically tailored to *S. rugosoannulata* remain limited, underscoring the necessity for innovative storage solutions.

Edible coating technology has emerged as a promising approach for mushroom preservation (Zhang et al., 2024). Among coating materials, chitosan (CS) has garnered widespread attention for its excellent film-forming ability, antimicrobial activity, and biodegradability (Jiang et al., 2012). However, the inherent hydrophilicity of CS represents a major limitation, compromising its efficacy in moisture regulation and structural maintenance. The incorporation of titanium dioxide (TiO₂) nanomaterials into CS (CS/TiO₂) has been demonstrated to enhance the physical and mechanical properties of the resulting composite (Surendhiran et al., 2022). Previous investigations have shown that CS/TiO₂ nanofilms are effective in mitigating oxidative processes and thereby delaying the postharvest senescence of *Agaricus bisporus* (Sami et al., 2021). A critical drawback of TiO₂, however, lies in its reliance on UV light for antibacterial activation, which restricts its applicability in typical postharvest storage environments. A promising strategy to overcome this limitation is nitrogen (N) doping, which extends the photocatalytic activity of TiO₂ into the visible light region. N is considered a suitable dopant due to its small atomic size, low ionization energy, high stability, and structural similarity to oxygen, enabling its incorporation into the TiO₂ lattice without altering the crystal structure (Chakraborty et al., 2023). While the preparation of CS/N-TiO₂ composites via co-precipitation has been previously reported for the degradation of patulin in apple juice (Huang & Peng, 2021), their application in mushroom preservation remains unexplored. Moreover, the combination of such nanocomposites with additional antimicrobial agents offers the potential for synergistic inhibition of the specific spoilage fungi associated with *S. bisporus*.

Against this background, the present study introduces the application of CS/N-TiO₂ composite films for the postharvest preservation of *S. rugosoannulata*. To further enhance preservation efficacy, four antimicrobial agents, natamycin (NT), nisin, ε-polylysine (ε-PL), and tea polyphenols (TP), were screened for their activity against the previously identified spoilage fungi and incorporated into the CS/N-TiO₂ matrix. A systematic evaluation was conducted on the microstructural characteristics and barrier properties (water vapor transmission rate, oxygen transmission rate, and mechanical strength) of the resulting composite films. Subsequently, the preservation efficacy of the optimized coating was assessed across three storage temperatures (4 °C, 10 °C, and 25 °C), with lightness (L*), weight loss rate, and decay rate employed as core quality indicators. We hypothesized that the preservation mechanism of the composite coating is temperature-dependent. To investigate this, correlation analyses were performed on respiratory intensity, total colony count, malondialdehyde (MDA) content, and the activities of catalase (CAT), peroxidase (POD), polyphenol oxidase (PPO), and superoxide dismutase (SOD). Furthermore, random forest risk ratio and Cox proportional hazards models were utilized for the prediction of storage period under varying conditions. The aim of this study is to realize the regulation of the optimal fresh-keeping effect of the composite film when storing *S. rugosoannulata* at different temperatures, clarify the differences in the mechanism of the composite film under different temperature scenarios, and provide a scientific theoretical basis and practical technical support for the efficient and long-term storage of *S. rugosoannulata* under multi-temperature gradients.

## 2 Materials and methods

### 2.1 Materials

*S. rugosoannulata* samples at the commercial maturity stage (with unopened pileus and intact veil), uniform in size and shape, white in stipe color, and free from mechanical damage, were obtained from a regional farm in Wuhan (Hubei Province, China), and transported to the laboratory within 6 h of harvest. CS (at deacetylation degree 90%, of food grade) was provided by Yusuo Chemical Technology Co., Ltd. (Shandong, China). TiO_2_ (primary particle size 35–75 nm) was purchased from Shaoxing Lijie Chemical Technology Co., Ltd. (Zhejiang, China). NT, nisin, ε-PL and TP (food grade) were purchased from Shandong Yuantaibio Engineering Co., Ltd. (Shandong, China), Hangzhou Jinlimeidi Biotechnology Co., Ltd. (Zhejiang, China) and Zhengzhou Bainaifo Bioengineering Co., Ltd. (Henan, China), respectively.

### 2.2 Preparation of CS/N-TiO₂ nanohybrid films

CS/N-TiO₂ nanohybrid was synthesised via the co-precipitation method (Huang & Peng, 2021). Briefly, 1.00 g of CS was dispersed in 100 mL of 1% (*v*/v) aqueous acetic acid solution and stirred continuously until a transparent solution was obtained. Then, 1.00 g of TiO₂ and 0.30 g of urea were added and thoroughly blended. The mixture was subjected to ultrasonication for 30 min, followed by magnetic stirring for 2 h to obtain a homogeneous milky-white suspension. The pH of the solution was adjusted to 10.0 by adding 1.0 M NaOH, resulting in the formation of a white precipitate. Cross-linking was induced by adding 0.40 mL of 25% glutaraldehyde under stirring for 10 min. After the reaction, the precipitate was washed with distilled water until neutral.

For the preparation of antimicrobial composite films, 1.00 g of each antibacterial agent (NT, nisin, ε-PL, and TP) was separately added to the above neutralized precipitate, followed by thorough stirring and degassing via ultrasonication for 30 min. Subsequently, 20 mL of each coating solution was cast into 9 × 9 cm circular plastic molds and dried at 60 °C for 4 h. Films without antibacterial agents, namely 1% CS and CS/TiO₂, were prepared under the same conditions to serve as controls.

### 2.3 Effect of films on mycelial growth of spoilage fungi from *S. rugosoannulata*

Spoilage fungi were isolated and purified from *S. rugosoannulata* as described previously (Liu et al., 2024). Four predominant spoilage strains were selected and identified as *F. pseudoanthophilum*, *A. niger*, *R. azygosporus*, and *T. purpureogenus* based on morphological and molecular biological analyses. The effects of the composite films on mycelial growth were evaluated according to the method of Yang et al. (2016) with minor modifications. Briefly, 2.0 mL of each composite film-forming solution was mixed thoroughly with 18.0 mL of sterile potato dextrose agar (PDA) medium cooled to approximately 45 °C, and the mixture was poured into sterile Petri dishes. After solidification, mycelial discs (5 mm in diameter) taken from the margin of actively growing colonies of each test fungus were inoculated at the center of each plate. The plates were incubated at 28 °C for 72 h. Colony diameters were measured in two perpendicular directions using the cross-streak method, and the average values were used for inhibition rate calculation. The mycelial growth inhibition (MGI) rate was calculated using the following formula:

(1) $$\mathrm{MGI}=\frac{\mathrm{dc}-\mathrm{dt}}{\mathrm{dc}}\times 100\%$$

where *dc* is the mean colony diameter of the control sets and *dt* is the mean colony diameter after 72 h.

### 2.4 Characterisation and physicochemical properties of composite films

#### 2.4.1 Characterisation

Fourier Transform Infrared (FT-IR) spectra were measured using a spectrometer (NEXUS-470, Nicoletnexus, Japan) in the wavelength range of 4000–400 cm^−1^. X-ray diffraction (XRD) patterns were determined using an X-ray diffractometer (D8 Advance, Bruker, Germany) and Cu—K as a radiation source. The surface microstructure was examined using Scanning Electron Microscopy (SEM, SU 8010, Tianmei, China).

#### 2.4.2 Mechanical properties

Film thickness was determined using a digital micrometre (accuracy: 0.001 mm, *n* = 10) (Aladdin, Shanghai, China). Mechanical properties were tested according to the method of Ni et al. (2022). Tensile strength (TS) and elongation at break (EAB) were calculated using the following equations:

(2) $$\text{Tensilestrength}\;\left(\mathrm{TS}\right)=\frac{F}{S}$$

where *TS* (MPa) is the tensile strength, *F* (N) is the maximum stress of stretching and *S* (mm^2^) is the sectional area of the films.

(3) $$\text{Elongation}\;\mathrm{at}\;\text{break}\;\left(\mathrm{EAB}\right)=\frac{L-L0}{L0}\times 100\%$$

where *EAB*(%) is the elongation at break, *L*_*0*_ (mm) is the initial length of films, *L* (mm) is the length at break.

#### 2.4.3 Barrier properties

Water vapor permeability (WVP) was measured using a method described by Lin et al. (2020) and calculated as follows:

(4) $$\mathrm{WVP}=\frac{∆m\times d}{∆t\times A\times ∆P}\;$$

where *WVP* (g·m^−1^·Pa^−1^·s^−1^) is the water vapor permeability, *∆m* (g) is the weight difference, *d* (m) is the thickness of films, *∆t* (s) is the permeation time, *A* (m^2^) is the permeation area of films and *∆P* (Pa) is the pressure difference on both sides of films at 25 °C.

Oxygen permeability (OP) and carbon dioxide permeability (CDP) were determined according to the Chinese National Standard GB/T 1038.1–2022 using a differential pressure gas permeation instrument (VAC—V2, Jinan Labthink Electromechanical Technology Co., Ltd., Shandong, China) (Jiang et al., 2024).

### 2.5 Application of films to mushrooms

*S. rugosoannulata* samples with no mechanical damage, uniform maturity and consistent color were selected and randomly divided into six groups. They were soaked with CS/N-TiO_2_/NT composite film solution for 60 s and then removed and dried naturally (hereinafter referred to as the film-coated group). The mushrooms soaked in ultrapure water for 60 s were used as the control (hereinafter referred to as the blank group). The storage test was carried out at 4 °C, 10 °C and 25 °C, respectively.

#### 2.5.1 Weight loss rate, color and decay rate

Weight loss rate of *S. rugosoannulata* was calculated using the following formula:

(5) $$\text{Weight loss late}\;\left(\%\right)=\frac{m0-m1}{m0}\times 100\;$$

where *m*_*0*_ is the fresh weight (g) and *m*_*1*_ is the weight after the sampling date (g).

Color parameters were assessed using a colourimeter (DR-410, KONICA MINOLTA, Japan) by measuring the lightness (L*) values of the mushroom samples (Duan et al., 2024). Decay rate was expressed as the percentage of decayed mushrooms. Samples showing black spots, surface mycelial growth, or soft rot were considered decayed.

#### 2.5.2 Total colony count, respiration rate, malondialdehyde (MDA) and enzyme activity tests

The total colony count was quantified using PCA plate medium (Wang et al., 2025). Incubation was conducted at 37 °C for 48 h, with results expressed as logarithmic values of colony-forming units per gram (log CFU/g).

MDA content was determined using a malondialdehyde content assay kit from the Nanjing Jiancheng Bioengineering Institute (Nanjing, China).

The activity of antioxidative enzymes, such as superoxide dismutase (SOD), catalase (CAT), peroxidase (POD) and polyphenol oxidase (PPO) was tested using respective assay kits according to the manufacture's instructions (Nanjing Jiancheng Bioengineering Institute, Nanjing, China). Briefly, 4 g of samples was grounded on ice using 36 mL of 50 mM phosphate buffer saline (pH 7.8) and then homogenised at 10000 ×g for 20 min at 4 °C. The supernatant was collected for the enzyme activity test.

### 2.6 Statistical analysis

The experiments were repeated three times, and statistical analysis was carried out using analysis of variance (ANOVA) and Duncan's multiple range test (*P* < 0.05) with SPSS Statistics 20 software. Spearman's correlation coefficient between the spoilage bacteria and the key metabolites was analyzed and visualized using the Oebiotech tools at https://cloud.oebiotech.cn/task. Analyses of the random forest risk ratio and Cox regression model were conducted using Python 3.9.7.

## 3 Results

### 3.1 Antifungal activity of composite films against spoilage Fungi

**Fig. 1:**
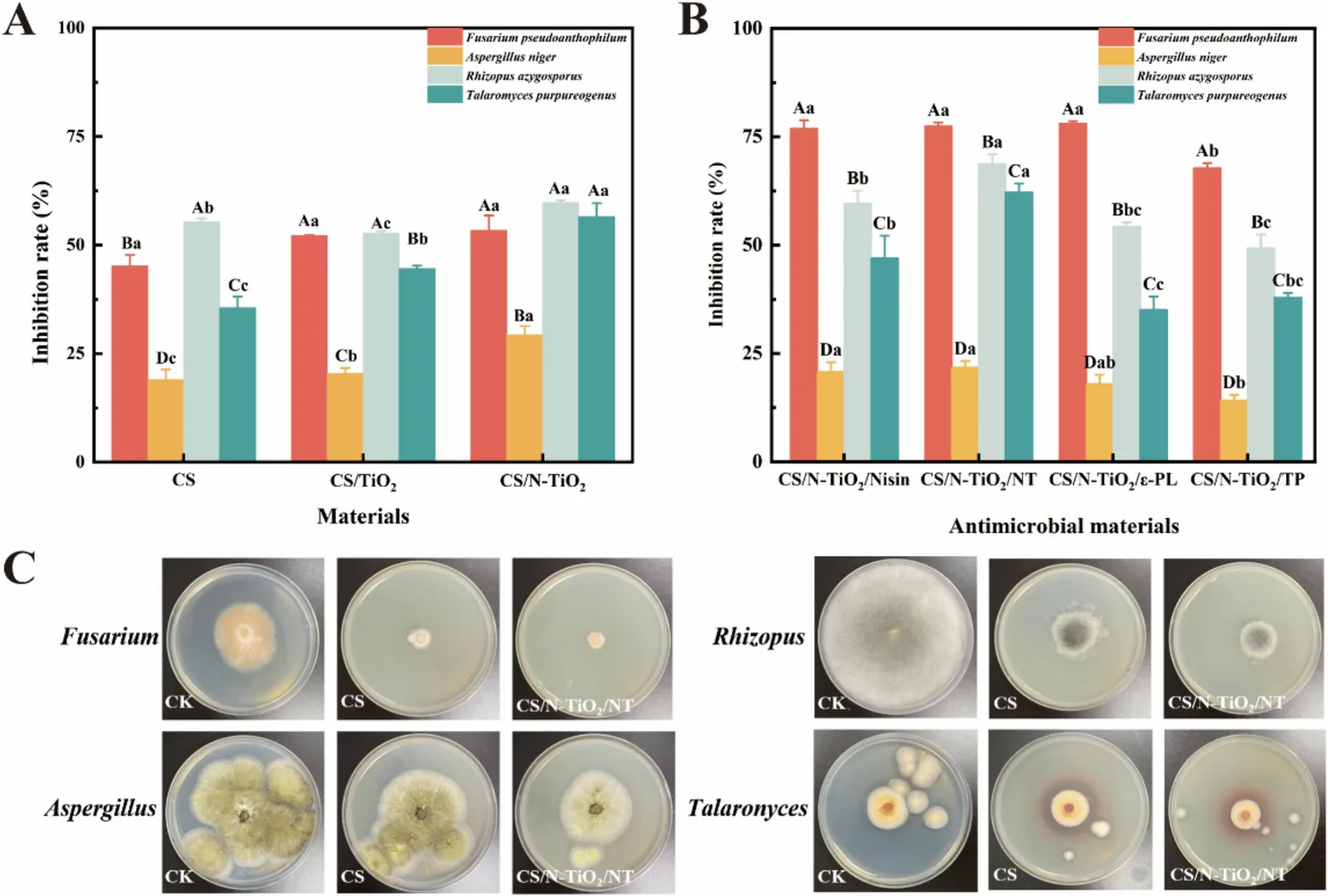
Inhibition rates of different film materials against the rot-inducing fungus from *S. rugosoannulata* (**A** and **B**) and the mycelial growth and colony morphology (**C**) of the rot-inducing fungus on different film materials.Different capital letters indicate significant differences among different spoilage fungi for the same material, while different lowercase letters indicate significant differences among different materials for the same spoilage fungus (*P* < 0.05).

The antifungal activity of various film formulations was evaluated against four spoilage fungi isolated from *S. rugosoannulata*: *F. pseudoanthophilum*, *A. niger*, *R. azygosporus*, and *T. purpureogenus* (Fig. 1A). The CS film exhibited moderate inhibition, with rates ranging from 18.98% against *A. niger* to 55.32% against *R. azygosporus*. Incorporation of TiO₂ into CS (CS/TiO₂) resulted in marginal, non-significant improvements against selected strains, whereas nitrogen-doped TiO₂ (CS/N-TiO₂) composites achieved significantly enhanced inhibition against *A. niger*, *R. azygosporus*, and *T. purpureogenus* (*P* < 0.05). Such enhancement stems from nitrogen doping-induced bandgap narrowing, extending photocatalytic activity into the visible-light region (Salzano et al., 2026) and enabling reactive oxygen species generation under typical storage illumination, a synergistic interplay between CS-mediated membrane compromise and ROS-driven oxidative damage underpinning the superior performance of CS/N-TiO₂ relative to binary counterparts.

Further enhancement was achieved by incorporating four antimicrobial agents, natamycin (NT), nisin, ε-polylysine (ε-PL), and tea polyphenols (TP), into the CS/N-TiO₂ matrix (Fig. 1B). All four composites achieved high inhibition against *F. pseudoanthophilum* and *A. niger*, with no significant differences among NT, nisin, and ε-PL groups. Against *R. azygosporus* and *T. purpureogenus*, however, the CS/N-TiO₂/NT composite demonstrated significantly superior inhibition compared to nisin- or ε-PL-containing counterparts (*P* < 0.05), whereas TP incorporation consistently yielded the lowest activity across all strains. Notably, the inhibition rates of nisin-, ε-PL-, and TP-containing composites against *A. niger* and *T. purpureogenus* were lower than that of CS/N-TiO₂ alone (Fig. 1B and Fig. 1A). This observation may be attributed to electrostatic repulsion between the cationic polypeptides (nisin and ε-PL) and the positively charged chitosan matrix, which could cause these agents to localize on the film surface and undergo premature loss, while the strong antioxidant properties of tea polyphenols may scavenge reactive oxygen species generated by N-TiO₂, thereby interfering with the antifungal mechanism.

The superior antifungal performance of the CS/N-TiO₂/NT composite was further corroborated by morphological observations (Fig. 1C). Relative to CS films, the ternary composite markedly restricted mycelial development, manifesting as reduced colony diameters and sparse, suppressed hyphal growth across all four fungal species.

### 3.2 Physicochemical characterisation of CS/N-TiO₂/NT composite film

#### 3.2.1 Structural characterisation

**Fig. 2:**
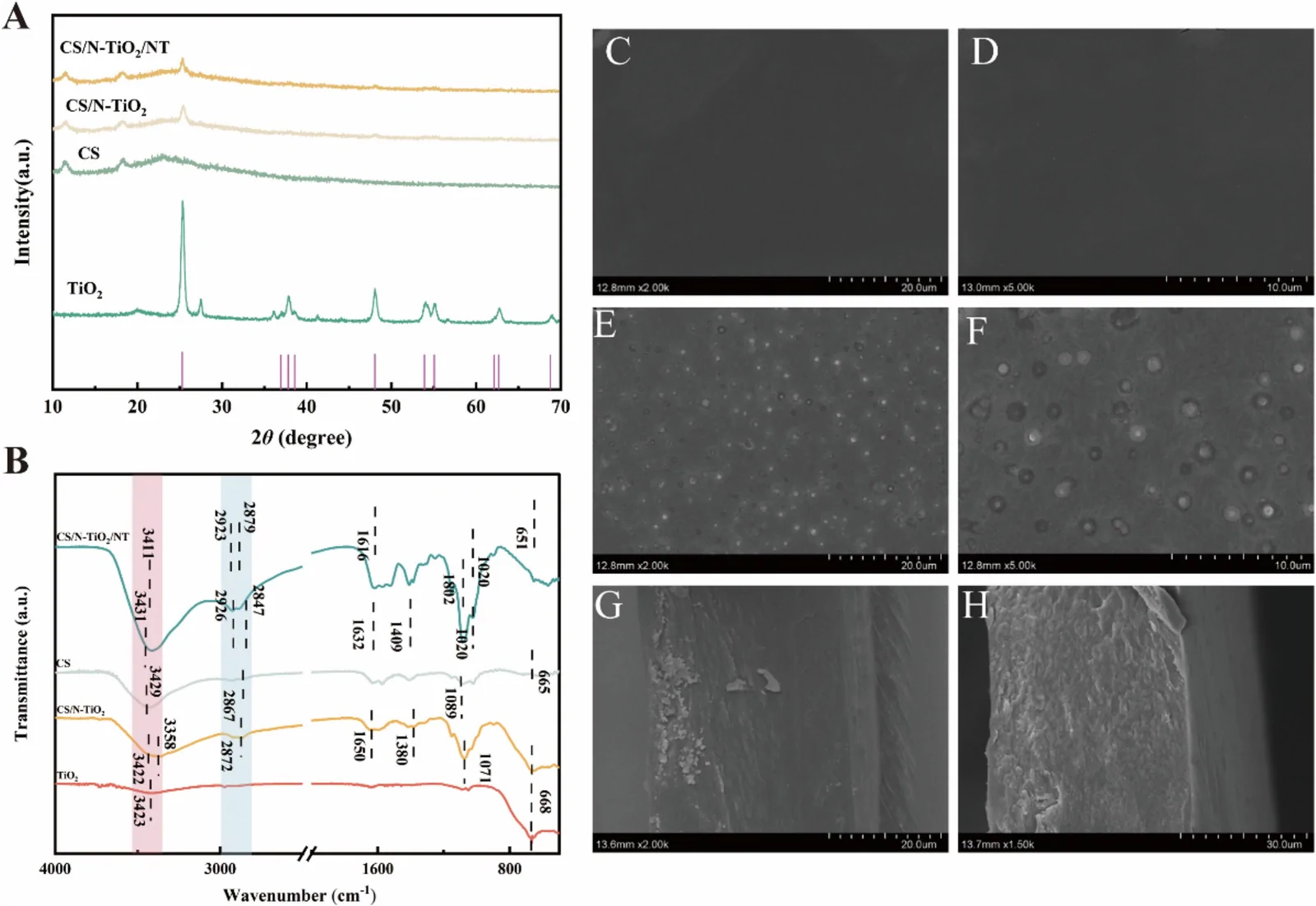
XRD pattern (**A**), FT-IR spectra (**B**), and flat (**C**, **D**) as well as cross-sectional (**G**, **H**) SEM images. Among them, **C**, **D** and G are images of the CS film, while **E**, **F** and **H** are images of the CS/N-TiO_2_/NT film.

As shown in Fig. 2A, pristine TiO₂ exhibited characteristic diffraction peaks at 2θ values of 25.3°, 27.4°, 37.8°, 48.09°, 55.18°, 62.96°, and 68.81°, consistent with the anatase crystalline phase. The CS film displayed four diffraction peaks at 8.4°, 11.5°, 18.3°, and 22.9°, where the former two correspond to the crystalline structure of CS and the latter two to its amorphous and crystalline components, respectively (Madian & Mohamed, 2020). Upon formation of the CS/N-TiO₂ composite, the characteristic peaks of both components were retained with reduced intensities, indicating successful incorporation of N-TiO₂ into the CS matrix without disruption of the original crystal structures. The CS/N-TiO₂/NT composite exhibited a similar diffraction pattern to that of CS/N-TiO₂, suggesting molecular dispersion of NT within the composite matrix rather than formation of a separate crystalline phase.

FT-IR spectra (Fig. 2B) provided evidence of intermolecular interactions among the composite components. Compared with the CS film, the CS/N-TiO₂/NT composite exhibited a red-shift of the hydroxyl stretching vibration from 3431 cm^−1^ to 3411 cm^−1^ and a shift of the amide-I band from 1632 cm^−1^ to 1616 cm^−1^, indicative of hydrogen bonding formation between CS and N-TiO₂. The characteristic Ti—O stretching band narrowed and shifted from 668 cm^−1^ to 651 cm^−1^, further confirming the interaction between TiO₂ nanoparticles and the CS polymer backbone. A new absorption peak appeared at 1802 cm^−1^ in the CS/N-TiO₂/NT composite, attributable to the C=O stretching vibration characteristic of NT (Chakravartula et al., 2020), confirming successful NT incorporation.

Surface and cross-sectional morphologies were examined by SEM (Figs. 2C–2H). The CS film (Fig. 2C, D, G) exhibited a smooth, compact surface with a densely packed cross-sectional structure, reflecting the homogeneous nature of the pure CS matrix. In contrast, the CS/N-TiO₂/NT composite (Fig. 2E, F, H) displayed a noticeably rougher surface morphology with uniformly distributed small protrusions corresponding to N-TiO₂ and NT aggregates. Notably, no cracks or pores were observed on the surface or cross-section, indicating the formation of an integrated, defect-free composite structure.

#### 3.2.2 Mechanical and barrier properties

**Fig. 3:**
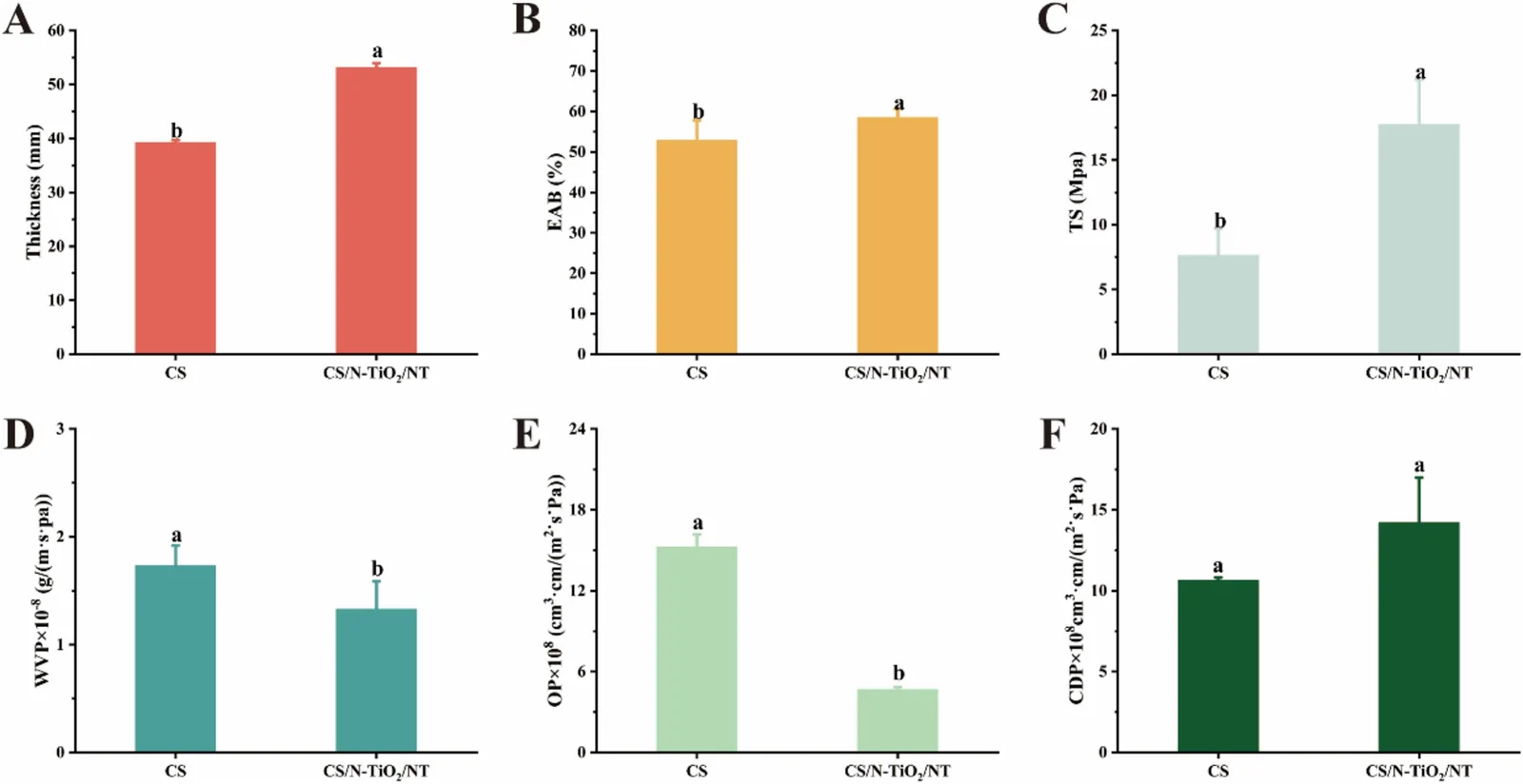
Thickness (**A**), elongation at break (**B**), tensile strength (**C**), water vapor permeability **(D**), oxygen permeability (**E**), and carbon dioxide permeability (**F**) of CS and CS/N-TiO₂/NT films.

As shown in Fig. 3A, the CS/N-TiO₂/NT composite exhibited a significant increase in thickness compared to the CS film (*P* < 0.05), with an average increment of 35.47%. The tensile strength (TS) of the composite increased by 132.20% relative to the CS film (Fig. 3C), a substantial enhancement attributable to the reinforcing effect of N-TiO₂ nanoparticles within the CS matrix. The formation of hydrogen bonds between N-TiO₂ and CS, confirmed by FT-IR analysis, establishes a stable three-dimensional polymer network that facilitates effective distribution of mechanical stress (Fig. 2 B). In contrast, the elongation at break (EAB) increased by only 10.69% (Fig. 3B), a change that did not reach statistical significance. This may be because the incorporation of N-TiO₂ enhances the film's strength while simultaneously restricting the mobility of chitosan chains, thereby limiting ductility. In addition, the incorporation of NT (natamycin) is known to reduce EAB (Sun et al., 2020). Notably, As illustrated in Fig. 3D, the water vapor permeability (WVP) of the CS/N-TiO₂/NT composite was significantly reduced (*P* < 0.05), a decrease attributed to two primary mechanisms: hydrogen bonding between N-TiO₂ and hydroxyl groups reduces the availability of free -OH groups in CS, thereby suppressing water molecule diffusion; additionally, the incorporation of N-TiO₂ nanoparticles results in a more compact film structure, prolonging the diffusion path for water vapor (Qian et al., 2011). Oxygen permeability (OP) exhibited a substantial reduction of 69.29% (Fig. 3E), indicating the composite's efficacy in restricting oxygen transmission, a favorable characteristic for suppressing oxidative browning and respiratory activity in stored mushrooms. In contrast, carbon dioxide permeability (CDP) showed no significant difference from the CS film (*P* ≥ 0.05) (Fig. 3F). This selective permeability pattern may facilitate the formation of a modified atmosphere around the packaged produce**.**

### 3.3 Preservation efficacy of CS/N-TiO₂/NT coating for *S. rugosoannulata* storage under variable temperatures

#### 3.3.1 Effects on quality attributes

**Fig. 4:**
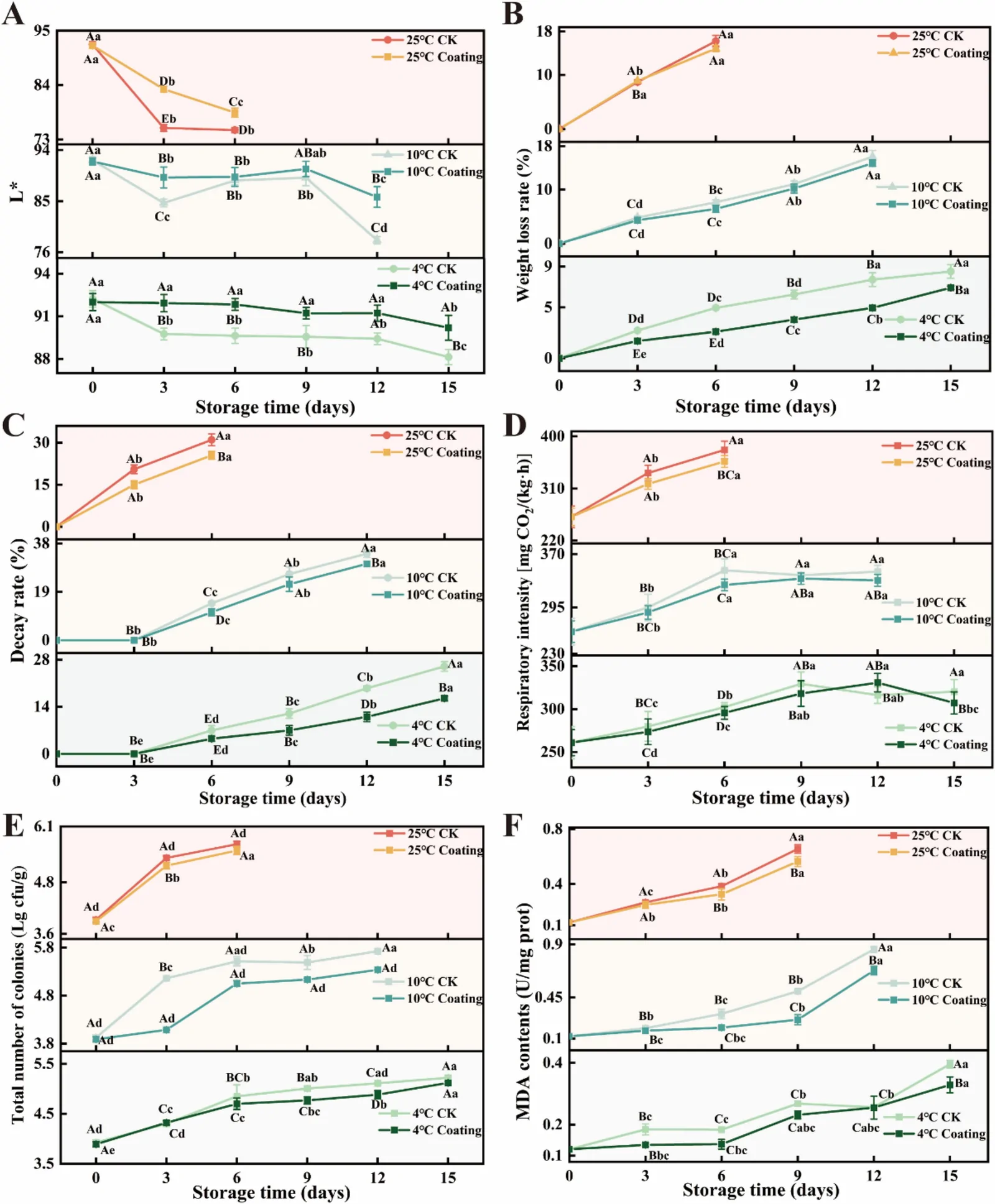
Changes in the quality parameters of *S. rugosoannulata* during storage. L*(**A**), weight loss rate (**B**), decay rate (**C**), respiratory rate (**D**), total colony count (**E**) and MDA (**F**). 4 °C CK, 10 °C CK and 25 °C CK: uncovered *S. rugosoannulata* stored at 4 °C, 10 °C and 25 °C, respectively. 4 °C Coating, 10 °C Coating and 25 °C Coating: coating-treated *S. rugosoannulata* with CS/N-TiO_2_/NT stored at 4 °C, 10 °C and 25 °C, respectively.

A progressive decline in L* values was observed across all storage conditions, indicating continuous browning of mushroom caps (Fig. 4A). At 25 °C, the control group exhibited rapid deterioration, with L* values dropping below 80 after 3 days, whereas the coated group maintained L* values above 80 throughout the same period (*P* < 0.05). The protective effect of the coating on color retention was more pronounced at 25 °C and 10 °C than at 4 °C, suggesting that the coating partially compensates for temperature-induced color deterioration. Weight loss increased progressively during storage, with higher temperatures accelerating moisture loss (Fig. 4B). The coated groups exhibited significantly lower weight loss rates than the control groups at both 4 °C and 10 °C (*P* < 0.05), with the most pronounced effect observed at 4 °C. This reduction reflects the effective moisture barrier properties of the composite film, as demonstrated by the reduced WVP values (Fig. 3 D). Decay rate, defined as the percentage of mushrooms showing visible spoilage, was substantially reduced by the coating treatment (Fig. 4C). At 4 °C on day 15, the control group exhibited a decay rate of 26.0%, while the coated group showed only 16.5%, representing a 36.54% reduction. This substantial decrease underscores the efficacy of the composite film in suppressing microbial spoilage during extended low-temperature storage.

#### 3.3.2 Effects on physiological and biochemical parameters

**Fig. 5:**
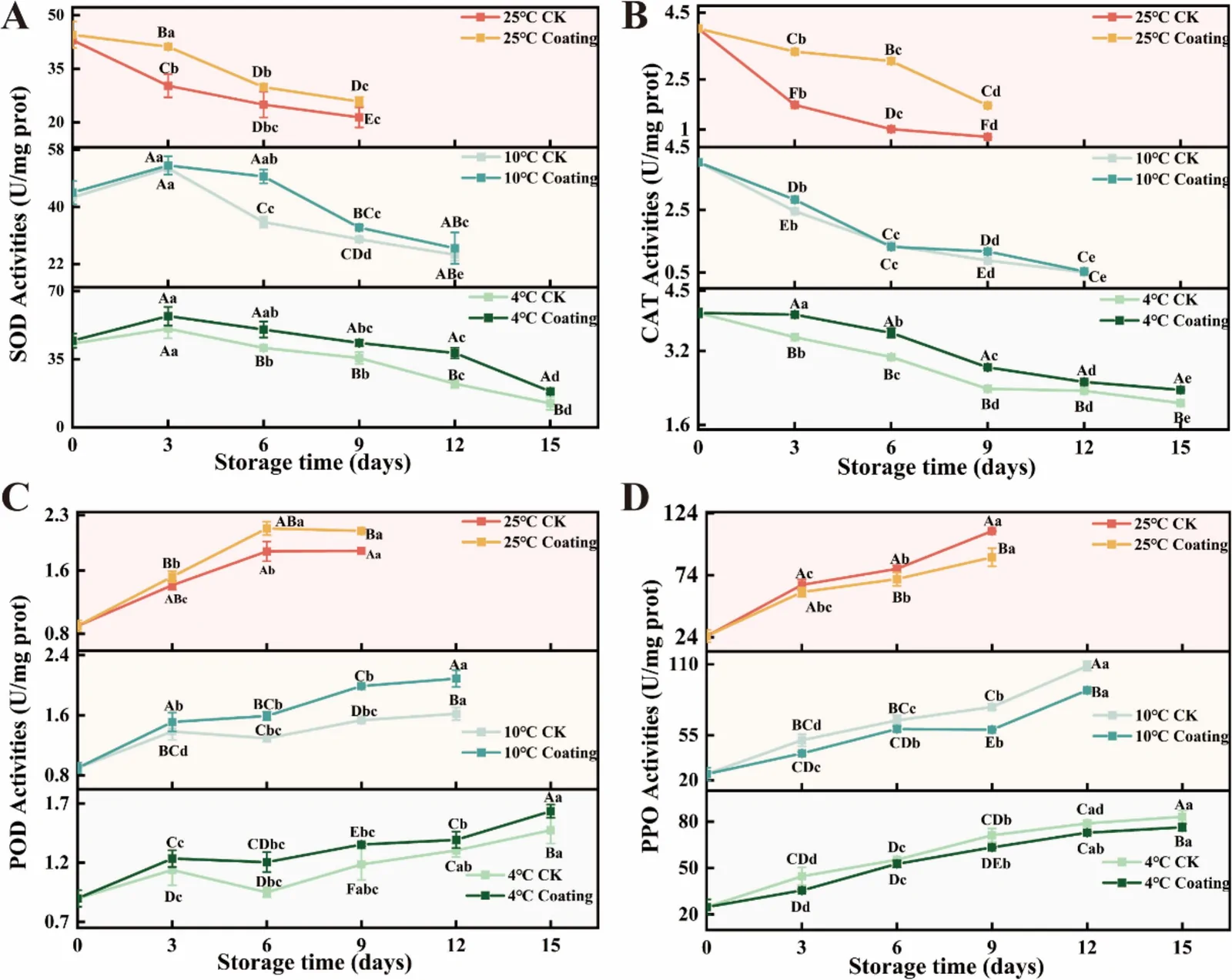
Changes in the enzyme activity of *S. rugosoannulata* during storage. SOD activity (**A**), CAT activity (**B**), POD activity (**C**) and PPO activity (**D**). 4 °C CK, 10 °C CK and 25 °C CK: uncovered *S. rugosoannulata* stored at 4 °C, 10 °C and 25 °C, respectively. 4 °C Coating, 10 °C Coating and 25 °C Coating: coating-treated *S. rugosoannulata* with CS/N-TiO_2_/NT stored at 4 °C, 10 °C and 25 °C, respectively.

The composite film suppressed respiratory activity, delaying the respiratory peak from 9 days to 12 days at 4 °C and from 6 days to 9 days at 10 °C (Fig. 4D). This suppression is consistent with the reduced OP of the composite film (Fig. 3E), which limits oxygen availability for aerobic respiration. Total colony counts were significantly reduced by the coating treatment, particularly at 10 °C (Fig. 4E). On days 3, 6, and 9 of storage at 10 °C, the coated groups exhibited reductions of 20.93%, 8.35%, and 6.56%, respectively, compared to the control groups. The pronounced antimicrobial effect at 10 °C reflects the temperature-dependent activity of the composite components, with moderate temperatures potentially facilitating the release of antimicrobial agents or enhancing their interaction with microbial cells. MDA content, an indicator of lipid peroxidation, increased progressively during storage (Fig. 4F). At 4 °C, no significant difference was observed between control and coated groups, whereas at 10 °C and 25 °C, the coated groups exhibited significantly lower MDA levels (*P* < 0.05), indicating that the composite film effectively mitigates oxidative damage under moderate and high temperature conditions. SOD activity showed an initial increase followed by a decline at 4 °C and 10 °C, whereas a continuous decrease was observed at 25 °C (Fig. 5A). The coated group maintained significantly higher SOD activity than the control group across all three temperatures, with the most pronounced effect at 10 °C, where the control group exhibited a significant decline after 6 days while the coated group maintained elevated activity until 9 days. CAT activity exhibited a decreasing trend throughout storage, with the coated group consistently showing higher activity than the control group, particularly at 25 °C (Fig. 5B). POD activity was activated by the coating treatment across all storage temperatures without altering the overall trend (Fig. 5C). The most substantial enhancement was observed at 10 °C, suggesting a temperature-dependent activation mechanism. At 25 °C, POD activity peaked at day 6 before declining, consistent with the onset of accelerated senescence. PPO activity increased continuously throughout storage, with the coated group exhibiting significantly lower activity than the control group at 10 °C and 25 °C (*P* < 0.05) (Fig. 5D). This reduction is consistent with the limited oxygen availability within the coated samples (Fig. 3E), as PPO-mediated browning requires oxygen as a substrate (Qin et al., 2015).

#### 3.3.3 Correlation analysis of quality and physiological parameters

**Fig. 6:**
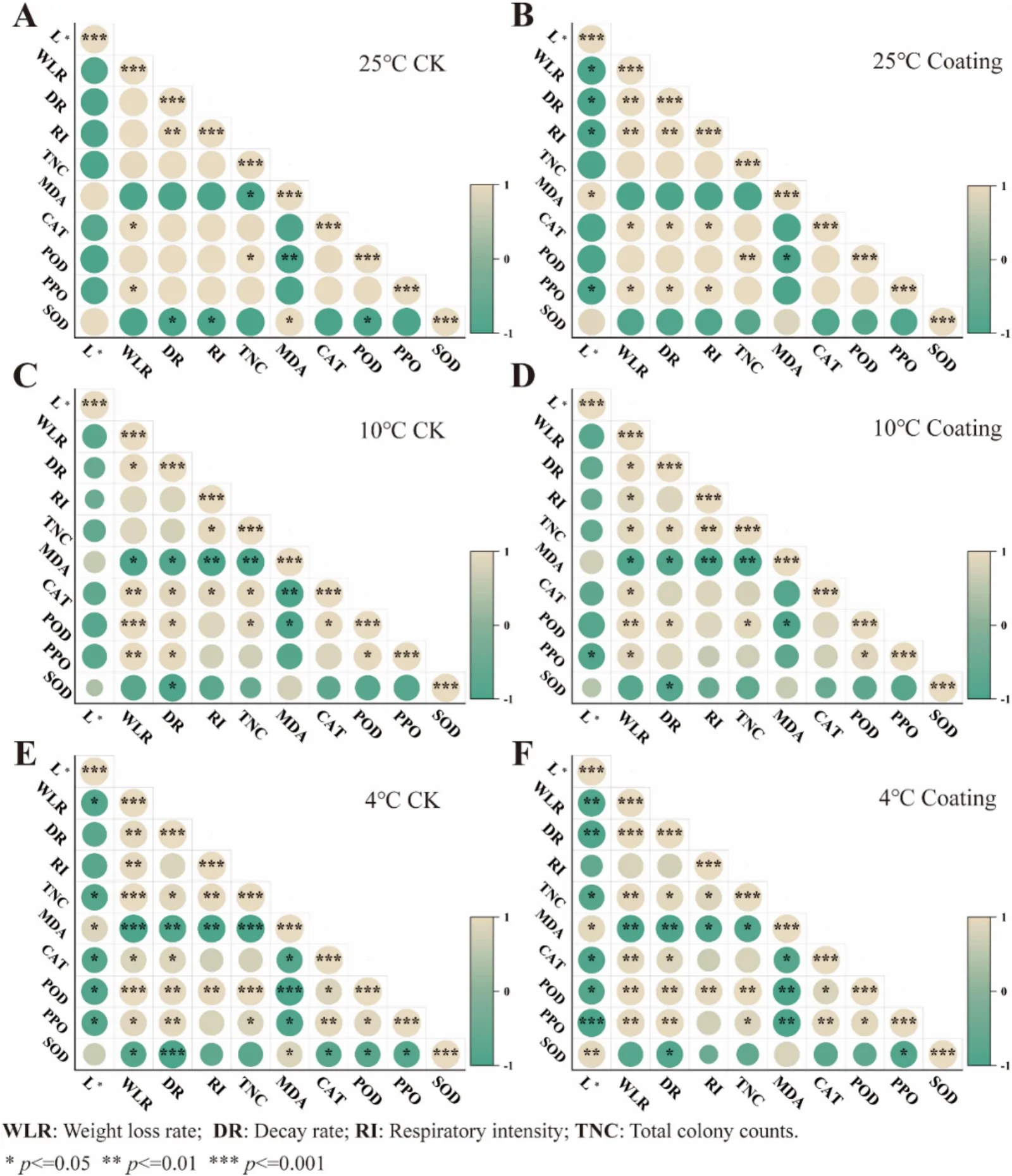
The correlations between key quality indicators (L^⁎^, weight loss rate, and decay rate) and physiological parameters (respiration rate, total number of colonies, MDA content, CAT activity, POD activity, PPO activity, and SOD activity).

To elucidate the relationship between quality deterioration and physiological responses, Pearson correlation analysis was conducted among key quality indicators (L* value, weight loss rate, decay rate) and physiological parameters (respiration rate, total colony count, MDA content, CAT, POD, PPO, and SOD activities). The results are presented separately for each storage temperature and coating condition in Fig. 6.

At 25 °C, no pronounced changes were observed between CK group and coating group (Fig. 6A, B). This finding suggests that, under high-temperature stress, the accelerated progression of senescence decouples quality attributes from the underlying metabolic and oxidative regulatory networks, with the coating treatment insufficient to restore such associations.

At 10 °C, distinct correlation patterns emerged between uncoated and coated samples (Fig. 6C, Fig. 6D). For uncoated mushrooms, quality parameters exhibited significant correlations with MDA content and the activities of CAT, POD, PPO, and SOD (*P* < 0.05), indicating a close association between oxidative stress, antioxidant enzymes, and quality maintenance at this moderate temperature. Notably, total colony count showed no significant correlation with quality in the uncoated group. Following coating application, the correlations between quality and POD, PPO, and SOD activities were substantially diminished, whereas total colony count became significantly correlated with quality (*P* < 0.05). This shift implies that, at 10 °C, the antimicrobial activity of the coating assumes a dominant role in quality preservation, effectively substituting for the oxidative regulatory mechanisms that governed quality in uncoated samples.

At 4 °C, the uncoated group (Fig. 6E, Fig. 6F) exhibited the most extensive correlation network, with quality parameters significantly correlated with respiration rate, total colony count, MDA content, and all four enzyme activities (*P* < 0.05). Such widespread correlations reflect sustained metabolic and oxidative activities during low-temperature storage. Coating treatment (Fig. 6F) markedly reduced these correlations, particularly for respiration rate, total colony count, and SOD activity, which no longer showed significant associations with quality. This selective decoupling indicates that the composite film mitigates quality deterioration through the inhibition of microbial growth, the suppression of respiratory activity, and the stabilization of SOD-mediated antioxidant defense.

#### 3.3.4 Storage life prediction using cox proportional hazards model

To further evaluate the effect of the composite film, the storage life prediction of *S. rugosoannulata* under different storage temperatures was conducted. After the feature importance evaluation using the random forest, the key core factors were determined as L*, PPO activity, temperature, MDA content, and coating treatment. The hazard ratios (HRs) were calculated through Model (6).

(6) $$h\left(t\right)=h_{0}\left(t\right)∙\exp\left(\beta_{1}\mathrm{PPO}+\beta_{2}L^{*}+\beta_{3}T_{10{}^{\circ}C+}\beta_{4}T_{25{}^{\circ}C}+\beta_{5}\mathrm{Coating}\right)$$

where *h(t)*: The spoilage risk rate at time t; *h₀(t)*: The baseline hazard function (for the untreated group at 4 °C); *T*₁₀ _°C_*, T*₂₅ _°C_: Dummy variables (with 4 °C as the reference, set to 1 for 10 °C and 25 °C respectively, and 0 otherwise); Coating: Coating treatment (1 indicates yes, 0 indicates no).

The event was defined as follows: When the spoilage rate was ≥30%, it was considered a failure (e.g., if the spoilage rate of the CK group was 31% on the 6th day, it was marked as the occurrence of an event).

With decay rate ≥ 30% defined as the endpoint event, storage life was determined using the Cox proportional hazards model (7).

(7) $$\text{Shelf Life}=\frac{\mathrm{Ln}\left(0.3\right)}{\lambda \cdot \exp\left(\beta_{1}\mathrm{PPO}+\beta_{2}L^{*}+\beta_{3}T_{10{}^{\circ}C+}\beta_{4}T_{25{}^{\circ}C}+\beta_{5}\mathrm{Coating}\right)}$$

The baseline hazard rate (λ) was calibrated using the untreated control group at 4 °C, with an empirically measured storage life of approximately 12 days (the decay rate of 30% observed between 13 and 15 days).

The predicted storage life indicated that, among the three storage temperatures, the 4 °C coating treatment demonstrated optimal effectiveness, with the potential to extend storage life by 33.33% to 38.46%. This percentage range was calculated based on the ratio of predicted storage life between coated and uncoated samples at 4 °C.

## 4 Discussion

### 4.1 Enhanced antimicrobial activity of the CS/N-TiO₂/NT composite

The preservation of postharvest mushrooms is critically challenged by their susceptibility to microbial spoilage, particularly given the absence of a protective cuticle. The ternary CS/N-TiO₂/NT composite developed in this study addresses this challenge through a multi-tiered antifungal mechanism. The electrostatic interaction between protonated amino groups of CS and the negatively charged fungal cell surfaces constitutes the primary mode of action, disrupting membrane integrity and inducing intracellular leakage (Li & Zhuang, 2020; Luo et al., 2025). However, CS alone exhibited limited efficacy against key spoilage fungi such as *A. niger* and *T. purpureogenus*, necessitating enhancement strategies.

Nitrogen doping of TiO₂ extended its photocatalytic activity into the visible-light region (Qian et al., 2011; Salzano et al., 2026). Under typical storage illumination, N-TiO₂ generates reactive oxygen species (ROS) that inflict oxidative damage on fungal cells, synergistically complementing the membrane-compromising action of CS. This synergy explains the significantly enhanced inhibition observed for CS/N-TiO₂ against *A. niger*, *R. azygosporus*, and *T. purpureogenus* relative to CS alone. NT exhibited good inhibitory effects on fungi as a biosourced antifungal substance. It inhibited fungal growth by binding to sterols in the cell membrane (Zhang et al., 2022). Fig. 1(B) showed that CS/N-TiO_2_, when combined with NT, enhanced the inhibition rates against *F. pseudoanthophyllum*, *T. purpureogenus* and *R. azygosporus* to varying degrees.

### 4.2 Structural reinforcement and barrier enhancement

Nanoparticles typically existed in water as unstable aggregates. Loading N-TiO_2_ onto the CS film facilitates their uniform dispersion and increases the specific surface area. Due to the electrostatic repulsion between the inorganic nanoparticles and the polymer matrix, the flexible chains of CS molecules were fully extended, improving the dispersion of nanoparticles within the polymer matrix. This provided the composite film with a stable mesh structure and a larger working area (Li et al., 2016). NT tends to form droplets on the fruit peel surface, leading to uneven distribution that adversely affects its antibacterial performance. The combination of NT and CS could leverage the film-forming property of CS to enable a more uniform distribution of NT on the sample surface (Zhang et al., 2022). Furthermore, the cross-section of the CS/N-TiO_2_/NT composite film exhibited a rougher texture. Similar structures have been observed in CS films loaded with glycerol monolaurate and nano-TiO₂ (Chang et al., 2021).

The physicochemical characterisation of the composite film was conducted to evaluate its potential applications. Film thickness is crucial for food preservation, and also influences its mechanical and barrier properties (Sun et al., 2020). Tensile strength (TS) represents the maximum tensile stress a film can withstand, while elongation at break (EAB) denotes the maximum change in length before fracture (Sun et al., 2020). The CS/N-TiO_2_/NT composite film exhibited increased thickness and enhanced mechanical properties, particularly in TS. This notable enhancement is consistent with the findings of Kaewklin et al. (2017). The observed phenomenon may be attributed to the reinforcement of the film's network structure resulting from N-TiO_2_ nanoparticles incorporation. However, Zhu et al. (2019) reported a significant decrease in EAB after the addition of TiO₂ to CS, whereas He et al. (2016) observed an increase. This discrepancy likely arises from differences in TiO₂ content, as an inappropriate loading may induce self-aggregation of the nanoparticles, thereby compromising the mechanical integrity of the composite film (He et al., 2016). Additionally, the incorporation of NT has been shown to reduce EAB (Sun et al., 2020). In the present study, the EAB of the CS/N-TiO₂/NT composite exhibited no statistically significant change relative to the CS film. This result demonstrates that, under the formulation and preparation conditions employed herein, the incorporation of N-TiO₂ and NT did not appreciably compromise film ductility, an outcome that is advantageous for practical food packaging applications. Elucidating the precise factors governing EAB in these composite systems will require further systematic investigation, particularly through concentration-dependent studies of N-TiO₂ loading.

Barrier properties are crucial for preserving food quality and extending product shelf life (Chang et al., 2021). The WVP, OP and CDP levels represent the ability of packaged food to exchange water vapor, oxygen and carbon dioxide with the surrounding atmosphere, respectively. Water and gas molecules are distributed on one side of the film and move through the spaces between polymer chain segments, eventually transferring to the other side via desorption (Zhu et al., 2019). Ideally, WVP should be as low as possible. The hydrophobicity of NT along with the insoluble inclusion compounds formed upon its addition, enhances the moisture resistance of the film. N-TiO_2_ may influence the microscopic network of the film and provided a tortuous path for water vapor (Karthikeyan et al., 2017). It may also interact with the hydrophilic –OH and –NH groups of CS, thereby reducing water vapor adsorption on the film surface. Similar research results were reported by Chang et al. (2021), who found that WVP was significantly reduced due to the hydrophobic substances glycerol monolaurate and TiO₂. The concentrations of oxygen and carbon dioxide had a significant impact on freshness and quality, and lower permeability to oxygen and carbon dioxide regulates respiration rate and extends shelf life (Zhang et al., 2019). Previous studies have indicated that incorporating hydrophobic substances into CS films typically leads to reductions in both CDP and OP. However, our findings revealed no significant difference in CDP and a marked decrease in OP. The underlying reasons for this divergent behavior demand further investigation, potentially involving the interfacial reorganisation of hydrophobic additives within the CS matrix and anisotropic molecular interactions.

### 4.3 Temperature-dependent preservation mechanisms of the CS/N-TiO₂/NT composite coating

The preservation efficacy of the CS/N-TiO₂/NT coating exhibited a pronounced temperature dependence, with distinct mechanistic pathways dominating at different storage temperatures. This temperature-dependent behavior is consistent with the correlation analysis results (Fig. 6) and storage life predictions (Table 1), which collectively demonstrate optimal preservation at 4 °C, intermediate efficacy at 10 °C, and limited effectiveness at 25 °C.

**Table 1:** Hazard ratios of key factors in the Cox model.

Variable	HR	Sig.	95% CI^d^
PPO	1.45	<0.001	1.32–1.59
L*	0.62	<0.001	0.55–0.70
T₁₀°C	2.10	0.002	1.52–2.90
T₂₅°C	4.78	<0.001	3.21–7.12
Coating	0.55	0.008	0.39–0.77

**Note:** 95% CI^d^ is the 95% confidence interval.

At 25 °C, the coating failed to establish significant correlations between quality parameters and physiological indices (Fig. 6A, B), consistent with the minimal preservation observed. High-temperature stress accelerates senescence to such an extent that metabolic and oxidative regulatory networks become decoupled from quality attributes, overwhelming the protective capacity of the coating (Zheng et al., 2022). Although the coating still reduced decay rate and delayed browning compared to controls (Fig. 4A, C), the overall preservation effect was marginal, indicating that temperature dominates over coating treatment under these conditions.

At 10 °C, the coating shifted the primary quality-determining factor from oxidative enzyme activities to microbial control. In uncoated samples, quality parameters were significantly correlated with MDA content and the activities of CAT, POD, PPO, and SOD (Fig. 6C), indicating that oxidative stress and antioxidant enzyme regulation were the dominant determinants of quality. Following coating application, these correlations were substantially diminished, while total colony count became significantly correlated with quality (Fig. 6D). This transition suggests that the antimicrobial activity of the composite assumes a dominant role at this temperature, effectively substituting for the oxidative regulatory mechanisms that govern quality in uncoated samples (Chang et al., 2021). The activation of POD activity (Fig. 5C) and the retardation of SOD decline (Fig. 5A) further support the role of the coating in modulating oxidative responses at 10 °C.

At 4 °C, the coating achieved optimal preservation through selective decoupling of quality from multiple deteriorative pathways. The uncoated group exhibited the most extensive correlation network (Fig. 6E), reflecting sustained metabolic and oxidative activities during low-temperature storage. Coating treatment markedly reduced correlations for respiration rate, total colony count, and SOD activity, which no longer showed significant associations with quality (Fig. 6F). This selective decoupling indicates that the composite film mitigates quality deterioration through three complementary mechanisms: (i) suppression of respiratory activity, as evidenced by the delayed respiratory peak (Fig. 4D) and consistent with reduced oxygen permeability (Fig. 3E); (ii) inhibition of microbial growth, reflected in reduced total colony counts (Fig. 4E) and decay rates (Fig. 4C); and (iii) stabilization of SOD-mediated antioxidant defense, evidenced by maintained SOD activity (Fig. 5A). Unlike at 10 °C, where antimicrobial activity became the dominant factor, at 4 °C the coating exerts a multi-target effect that simultaneously addresses physical, microbial, and oxidative deteriorative pathways. This multi-target mechanism explains the superior preservation efficacy at 4 °C, where storage life was extended by 33.33–38.46% compared to uncoated controls (Table 2).

**Table 2:** Storage life prediction using the Cox proportional hazards model.

Condition	Predicted storage life (days)	Verified with actual measurement data
4 °C-coating	18–20	Decay rate was 16.5% and L* was 90.19 on the 15th day. (high freshness)
4 °C-CK	13–15	Decay rate reached 26.0% on day 15, approaching the critical value.
10 °C- coating	10–12	Decay rate on day 12 was 30%, consistent with model prediction.
10 °C-CK	8–9	Decay rate reached 26% on day 9 (approaching 30%) and exceeded 30% by day 12, demonstrating model effectiveness within acceptable risk control parameters.
25 °C- coating	≤ 6	Decay rate on day 6 was 25.5%
25 °C- CK	≤ 5	Decay rate on day 6 was 31.0%

The temperature-dependent hierarchy of preservation mechanisms, antimicrobial dominance at 10 °C versus multi-target action at 4 °C, reflects the interplay between coating functionality and temperature-modulated physiological responses. At moderate temperatures (10 °C), microbial proliferation becomes a primary driver of quality loss, and the coating's antimicrobial properties emerge as the dominant protective mechanism. At low temperatures (4 °C), microbial growth is inherently suppressed, allowing the coating's physical barrier properties (reduced WVP and OP) and antioxidant-stabilizing effects to contribute more substantially to quality retention. This mechanistic hierarchy underscores the importance of temperature management as a critical determinant of coating efficacy.

## 5 Conclusion

This study successfully fabricated a CS/N-TiO₂/NT composite film and demonstrated its efficacy in extending the postharvest storage life of *S. rugosoannulata*. Mechanistically, nitrogen-doped TiO₂ extends photocatalytic antimicrobial activity into the visible spectrum, while natamycin provides targeted fungicidal effects, synergistically reinforced by the film-forming and barrier functions of chitosan. The concerted action of these components substantially enhances the overall preservation efficacy. Furthermore, the preservation efficacy of the coating was highly temperature-dependent. At 25 °C, protection was marginal due to accelerated senescence. At 10 °C, the primary determinant of quality retention shifted from endogenous oxidative enzyme regulation to microbial control, highlighting the dominance of antimicrobial functionality. Optimal preservation occurred at 4 °C, where the coating exerted multi-target effects, suppressing respiration, inhibiting microbial proliferation, and stabilizing SOD-mediated antioxidant defense. Cox proportional hazards model analysis revealed a 33.33–38.46% extension in storage life for coated samples at 4 °C compared to uncoated controls.These findings establish the CS/N-TiO₂/NT composite as an effective, temperature-responsive preservation strategy for perishable mushrooms, providing both a practical solution and a mechanistic framework for the rational design of postharvest interventions under varying storage temperatures.

## CRediT authorship contribution statement

**Xingjun Lu:** Writing – original draft, Methodology, Conceptualization. **Kun Qiao:** Writing – original draft, Methodology, Conceptualization. **Xinyan Liu:** Resources, Investigation. **Xiaozhen Peng:** Writing – review & editing. **Bangzhu Peng:** Supervision, Resources.

## Funding

This work was supported by Project of Hunan Provincial Research and Innovation Platform for Universities: Decoding of Novel Biomolecules in Liver Cancer and Intelligent Diagnosis(2025), and Edible Fungi Industry Chain Project of Hubei Province of China (NYCYL-2024-35).

## Declaration of competing interest

The authors declare that they have no known competing financial interests or personal relationships that could have appeared to influence the work reported in this paper.

## Data Availability

Data will be made available on request.
